# The qualitative experience of sexuality in ageing women: a narrative review

**DOI:** 10.3389/fgwh.2026.1793107

**Published:** 2026-03-13

**Authors:** Clement Boutry, Naomi Thorpe, Danielle De Boos

**Affiliations:** 1 Institute of Mental Health, School of Medicine, University of Nottingham, Nottingham, United Kingdom; 2 Knowledge and Library Services, Nottinghamshire Healthcare NHS Foundation Trust, Nottingham, United Kingdom

**Keywords:** ageing women, later-life sexuality, narrative review, psychosexual health, qualitative synthesis, sexual wellbeing

## Abstract

Sexuality remains an important yet frequently marginalised aspect of ageing women's health. Existing research has largely prioritised biomedical decline and menopausal dysfunction, often overlooking women's lived, relational, and sociocultural experiences of sexuality in later life. This narrative review synthesises qualitative evidence exploring how women aged 45 years and over experience, negotiate, and make sense of sexuality as they age. A targeted literature search identified nine qualitative studies, whose findings were thematically synthesised using a combined deductive–inductive approach. Analysis was organised around organic and physical factors, psychogenic and sociocultural influences, and support or intervention-related domains. Across studies, sexuality emerged as an enduring and adaptable aspect of identity rather than something that disappears with age. Women described navigating bodily change, chronic illness, and medication effects alongside powerful social forces, including ageism, stigma, gendered caregiving responsibilities, and healthcare invisibility. Sexual expression was frequently reframed through intimacy, relational connection, and solo practices rather than penetrative sex alone, and women highlighted evolving concerns such as risk of sexually transmitted infections in midlife and later life. The review identifies substantial barriers to accessing appropriate sexual health support and points to promising psychosocial and embodied approaches that may support confidence, sensuality, and wellbeing. Collectively, these findings challenge reductive biomedical models of sexual ageing and underscore the need for integrative, gender-sensitive approaches that recognise sexuality as a vital component of healthy ageing.

## Introduction

Ageing is a major demographic reality with profound health and social implications. In the UK, over a third of the population is aged 50+ (1), and by 2050 those aged 65+ are projected to comprise 16% of the population (2), highlighting the urgent need to understand and support healthy ageing.

Chronological definitions of ageing (commonly 60+) oversimplify the diversity and experiences of later life, shaped by health, lifestyle, genetics, and environment. The World Health Organization (WHO) advocates focusing on maintaining functional ability, or people's capacity to do what they value (3). England's National Health Service (NHS) has adopted this model, emphasising wellbeing and independence rather than decline (4, 5). This shift invites broader questions about what constitutes healthy ageing. Sexuality is a central yet often overlooked dimension which encompasses biological, psychological, and social elements shaping desires, intimacy, relationships and identity (6). The WHO defines sexual health broadly, including gender role and identity, orientation, pleasure, intimacy, and reproduction (7). Despite this, post-menopausal women are routinely depicted as asexual or invisible (8, 9), and when acknowledged, often portrayed as anomalies (10). Such stereotypes overlook evidence that sexual activity often continues into later life and contributes significantly to quality of life (11–13). Qualitative studies further link sexual satisfaction to perceptions of ageing well (14, 15), including women-focused research (16).

However, older women's sexuality is often shaped by broader social and cultural forces. Post-reproductive women are often framed as asexual, irrelevant or undesirable, reinforcing associations between youth and sexual value (17), neglecting older women's sexual health and rights. Because these influences and healthcare structures vary across countries, this review focuses on UK-based qualitative studies. The WHO (18) identified ageism as the third most common global discrimination with an urgency to dismantle age-based stereotypes. Older women's marginalisation is compounded by intersectionality such that ethnicity, class, disability, religion, and geography affect opportunities for expression and access to care (19, 20). Silence and taboo deter help-seeking (21), while some women resist by reclaiming agency and visibility, underscoring the need for inclusive, culturally sensitive perspectives of female sexuality across lifespan (22).

When older women are included in sexuality research, their experiences are often reduced to menopause and biology (23). Health and chronic illness complicate sexual wellbeing (24). Ageing increases the risk of chronic conditions such as diabetes, Parkinson's disease, and urinary incontinence interfering with sexual interest, dysfunction and satisfaction (25, 26). Medications for these conditions add barriers by impairing arousal, lubrication, and orgasm (27). Menopause compounds these issues: declining oestrogen causes vulvovaginal atrophy, vaginal dryness and dyspareunia (28, 29) adding to vasomotor symptoms (e.g., hot flashes), and contributing to insomnia, cognitive impairment, and depression; all of which diminish sexual function (29–31). Collectively, these changes reinforce enduring stereotypes of older women as “diseased”, “hormonal”, or “asexual” (32).

In response to these challenges, a range of interventions have been developed. Hormone replacement therapy (HRT) addresses menopausal symptoms (33), while lubricants and moisturisers provide non-hormonal relief (34). Aesthetic procedures such as vaginal laser therapy show potential, but lack robust evidence (35). Psychotherapeutic approaches, including CBT and mindfulness, may improve body image, desire, and anxiety (36, 37). Relationship-based techniques, such as Sensate Focus, support couples in adapting to changes in sexual function (38). Yet, barriers remain: stigma, healthcare systems that overlook sexual health beyond reproduction, and the scarcity of specialist services limit access. Research also tends to isolate organic and psychogenic factors rather than adopting a holistic and integrative approach (39).

Altogether, sexuality in later life remains central to wellbeing but is undermined by intersecting biological, psychological, and sociocultural challenges. Despite increasing recognition of its importance, women's voices remain underrepresented, interventions are unevenly accessible, and stigma persists. This review aims to address these gaps by examining the organic and psychogenic factors influencing older women's sexual health and exploring interventions that may support sexual wellbeing.

### Terminology note

Across the literature, terms such as midlife, older/later life, 50+, and postmenopausal are used inconsistently and do not map neatly onto one another. In this review, we use midlife and later life as an umbrella framing for women in later adulthood, while retaining study-specific age thresholds and menopausal descriptors where reported. We note that menopausal stage and chronological age overlap conceptually but are not synonymous.

## Method

Given the exploratory and integrative nature of the topic, a narrative review was selected to support theory-informed synthesis across diverse domains (e.g., biopsychosocial and treatment-related influences). A search of the literature was conducted by an Information Specialist in two healthcare databases (Ovid MEDLINE and Ovid PsycINFO). Search terms were organised around four core concept areas, using free-text terms (title/abstract) and controlled vocabulary where applicable: (1) sexuality/sexual health; (2) ageing and later life (including menopausal transition); (3) women; and (4) qualitative research. A UK filter was applied to ensure health-system and sociocultural specificity. Searches were limited to English-language papers published from 2015 onwards and to studies involving women aged ≥45 years. Full search strategies are provided in Supplementary Material A1.

Studies were included if they focused on women aged 45 and above, commonly defined as midlife to older adulthood, including postmenopausal populations in recent studies (40). Study populations were variously described as midlife, older/later life, 50+, or postmenopausal; we retained authors’ terminology and did not treat these categories as interchangeable unless explicitly defined by the study. Only primary qualitative studies examining the psychosexual impact of aging were included, given their ability to capture lived experiences, perceptions, and nuanced psychosocial factors. Review articles were screened for relevant theoretical or clinical insights. To ensure contemporary relevance in light of evolving sociocultural attitudes, clinical practices, and treatment approaches, only studies published from 2015 onward were included (41). Furthermore, only UK-based studies were selected to account for cultural and healthcare-system-specific influences on women's experiences (42). The term “women” refers to individuals identified as female in the included studies, including biological and self-identified women, where reported.

Studies were excluded if they focused on men or mixed-gender populations without disaggregated female data, involved couples without a female-specific focus, or addressed non-psychosexual topics without clear relevance to sexuality or body image. For mixed-gender studies that were included, we extracted and synthesised only findings explicitly attributable to women (i.e., author-reported gender-stratified themes/subthemes and/or quotations clearly identified as women's accounts); gender-aggregated findings were not extracted. Non-English publications, non-peer-reviewed materials (such as dissertations, grey literature, editorials, letters, and conference abstracts), and articles unavailable in full text were also excluded. Findings of selected articles were synthesised into three themes and then categorised into meaningful subthemes which in return further informed and refined the overarching themes.

A narrative thematic synthesis was undertaken focusing on the findings sections of the included qualitative studies. Data extraction prioritised authors’ reported findings alongside illustrative participant quotations (43). Analysis followed a combined deductive–inductive approach (44). Initially, findings were organised using three sensitising categories informed by existing literature: organic factors (e.g., hormonal changes, biological processes, medication side effects), psychogenic (e.g., body image, self-esteem, psychological adaptation), and treatment approaches (e.g., hormone therapy, aesthetic interventions, Psychosexual and Relationship Therapy (PRT). This framework was selected because it offers a clinically meaningful structure for integrating heterogeneous qualitative findings while remaining consistent with established biopsychosocial conceptualisations of sexual functioning (45, 46). These categories were used as a conceptual framework to guide the synthesis and analysis, providing a basis for identifying broad, integrative themes across studies. In the second stage, extracted material within each category was compared across studies to identify recurring patterns, tensions, and areas of divergence. These patterns were iteratively clustered and refined into subthemes that captured shared experiential processes across the nine studies. Importantly, while the initial categories provided structure, the final subthemes were allowed to transcend these boundaries, reflecting the interconnected and lived nature of ageing women's sexual wellbeing.

This approach prioritised interpretive integration rather than aggregation, aiming to illuminate how sexuality is experienced, negotiated, and reframed in later life. As such, the synthesis foregrounds women's subjective meanings and relational contexts rather than prevalence or causal inference (47). Due to the narrative review design, no formal quality appraisal or meta-analysis was conducted, which may limit the assessment of study quality and introduce potential selection bias. Formal quality appraisal was not undertaken because the review aimed to map and interpret recurring concepts in women's accounts (rather than weight evidence), and methodological rigour was addressed through restrictive eligibility criteria (peer-reviewed primary qualitative UK studies, full text available) and transparent screening and synthesis procedures.

The synthesis was conducted by an interdisciplinary team (psychology researcher/counsellor and trainee psychosexual therapist; clinical psychologist), with database searching supported by an information specialist. We recognise that our clinical/psychological positioning shaped attention to lived experience, meaning-making, and barriers to care, and we therefore kept interpretation closely anchored to the included studies throughout theme development.

## Results

The search identified 270 records. After duplicate removal, 185 unique records remained for screening. Following title and abstract screening, 39 articles were reviewed in full text; 30 were excluded (most commonly because they were not UK-based or did not meet the inclusion criteria). Nine studies met eligibility and were included in the synthesis (study characteristics: Supplementary Material A2). Data collection methods included semi-structured interviews (*n* = 6) and free-text responses (*n* = 3). Most studies focused exclusively on women; four included participants of different genders, but only findings relevant to women's experiences were extracted and synthesised.

Three overarching themes describing ageing women's experiences were identified: (1) organic and health-related dimensions of sexuality, (2) psychogenic and sociocultural influences, and (3) interventions and barriers to care.

### Theme 1: organic & health-related dimensions of sexuality in later life

Women's sexual wellbeing in later life was closely shaped by health and bodily changes associated with ageing. Menopause often marked a turning point, bringing bodily changes and reduced libido. Chronic illnesses and medication side effects further shaped desire and sexual function, while partner health facilitated or hindered intimacy. Alongside biological aspects, women reflected on body image, physical fitness, and energy levels, while also recognising evolving risks such as Sexually Transmitted Infections (STIs). Together, these organic and health-related factors illustrate how physical ageing intersects with sexuality.

#### Menopause and physical consequences

Menopause was a turning point in women's sexuality, with symptoms making penetrative sex “incredibly painful” (48–50). This led many women to seek help, as they felt they had lost “an extremely important part of their life: intercourse” (48). However, this impact was sometimes dismissed by healthcare professionals, who attributed sexual difficulties to age rather than menopause itself, reflecting the assumption that post-reproductive women's sexual health holds a lower priority (51).

Many women described adapting sexual practices in response to pain and menopausal change, often by drawing more on non-penetrative intimacy [see Theme 2; (52)]. Yet because this form of intimacy depends on having a partner, single women had fewer opportunities for sexual or affectionate expression.

#### Medical conditions and relational health barriers

Health conditions, rather than age, posed the main obstacles to sexuality, affecting both women and their partners and often compounding when multiple issues coexisted (49, 53). Reported difficulties included women's medical conditions, treatment side effects, and partners’ sexual dysfunctions. In some cases, medication directly reduced libido or contributed to male partners’ erectile difficulties, which were commonly cited as limiting sexual satisfaction in older women (49). Partners refusing medical support caused further frustration, leaving women feeling stuck (49). Fatigue was a commonly reported barrier, reducing energy for a regular and satisfying sex life (49, 53).

While often framed as a physical factor, it often stemmed from the competing demands placed on middle-aged women, a theme explored in the next section.

#### Physical appearance and body image

Women described a profound sense of loss related to ageing, including changes in body shape, weight, and skin, which contrasted with the youthful appearance they once took for granted.

Ageing was often seen as a passive process beyond their control, creating a gap between their subjective age (the age they felt inside) and bodily appearance (51, 54). Many equated “successful ageing” with maintaining youth, prompting comparisons with others (54) and reinforcing societal associations between femininity, youth, and beauty (52). While sexual desire was not always reduced, these physical changes affected self-esteem and confidence, bringing hesitation to initiate sexual activity (52).

#### STIs and sexual risks in later life

Menopause and the loss of pregnancy risk were sometimes seen positively, allowing reduced condom use and lower prioritisation of safer sex practices (49, 55). Several women reported feeling “immune” to STIs, leading to riskier behaviours such as not using condoms, skipping STIs testing, or relying on assumptions about partners based on histories or appearance (55).

In new relationships, condom use could feel awkward to introduce, particularly after long periods in monogamous partnerships. Resentment about initiating these conversations, along with social pressures to re-partner and awareness of younger family members’ relationships, shaped sexual decision-making. Long-term monogamy or a self-identity as a “relationship person” reinforced beliefs that protection was unnecessary, while for some, STI risk was considered only when trust was broken; others delayed testing until a relationship ended, leaving notable gaps in protection. Fears of ageism and judgment when purchasing condoms or seeking advice in non-sexual health settings further discouraged protective behaviours. Broader barriers to help-seeking and service access are addressed in Theme 3.

### Theme 2: psychogenic and sociocultural impact

Sexuality was deeply shaped by self-perception, relationships, and wider cultural messages. Ageing often prompted women to renegotiate sexual identity, balancing feelings of invisibility with resilience and self-acceptance. Gender roles and relational expectations positioned women as prioritising partners’ needs, while societal stereotypes of older people as asexual contributed to silence and stigma. At the same time, participants expressed agency, redefining intimacy as affection, closeness, or self-pleasure, even when partnered sex was difficult. This underscores the complex psychosocial context in which older women negotiate sexuality.

#### Sexuality as a persistent and adaptable self

While some women accepted the absence of sexual activity (49, 55), sexuality remained a valued aspect of identity in later life. For many, sexual expression extended beyond penetrative intercourse to encompass appearance, femininity, and desire, seen as natural elements of ageing well and maintaining health. Some reported feeling increasingly uninhibited with age, viewing sexual expression as a marker of vitality regardless of relationship status (51). Sexual practices were often adapted to health or relational circumstances, with women prioritising intimacy, physical closeness (51, 52), or solo activity (48). These accounts support the idea of a subjective sexual self-persisting across lifespan, independent of chronological age or societal stereotypes of older women's sexuality (54).

#### Social erasure and cultural invisibility

Despite growing openness around women's sexual health, this shift has largely excluded older women (51). Participants described pervasive stereotypes portraying older people as asexual, limiting their ability to express needs or pursue new relationships (52, 54). Media and dating platforms reinforced this invisibility, often reducing sexually active older women to caricatures such as “cougars” or highlighting only glamorous, youthful celebrities; portrayals experienced as unrealistic and subjecting older women to scrutiny (51, 55). Cultural depictions of later-life sexuality as deviant, distasteful, or trivial (a “peck on the cheek”) further undermined self-image (55). Such representations reinforced the notion that sexuality is for the young, linking “successful ageing” to youthful looks.

These cultural scripts were mirrored in care pathways (see Theme 3), where women anticipated judgement and invisibility when seeking support.

#### Competing demands and emotional burden

Conflicting demands, such as financial pressures, work, and caregiving, affected women's sexuality. For this generation's ageing women, these often translated into “double caring duties,” supporting both children/grandchildren and ageing parents, a pattern shaped by later childbearing and increased longevity (49, 52, 53, 55). Such responsibilities consumed energy and time, limiting sexual intimacy and discouraging the pursuit of new relationships (52). Gendered expectations extended care to partners, reinforcing prioritisation of others’ needs (55). Alongside these external pressures, women described internal struggles, including depression, bereavement, anxiety, and trauma, which further lowered self-esteem and sexual desire (55).

#### Emotional negotiations of desire and intimacy

Women reported diverse emotional responses to changes in sexual intimacy. Guilt arose in multiple ways: women felt guilt entering casual relationships after partner loss, over diminished desire or inability to engage in sex, worrying that partners expected more (52). Several continued sexual activity primarily to please their partner despite their own lack of satisfaction, reflecting internalised expectations to meet male sexual needs (48, 49). Women reported sadness over changes in sexual behaviour and feelings of loss (48), along with anxiety when reduced desire came from male partners, challenging the stereotype that men always want more sex (52). Frustration commonly arose when partners avoided discussing or addressing sexual difficulties, sometimes straining relationships (48, 52). However, many women reframed intimacy around affection, humour, care, and emotional closeness, showing that physical intimacy could remain meaningful even without intercourse (48, 49).

#### Relational contexts of sexuality

Relationships, or their absence, were central to women's experiences of sexuality in later life. New partnerships sometimes offered opportunities to feel more uninhibited and develop healthier sexual connections than earlier relationships (52, 53).

However, many women reported sexual inactivity due to widowhood/divorce, or lack of opportunity, often compounded by low confidence and anxieties about a changed dating landscape (49, 55). While some longed for intimacy (52), others were content without sex or saw monogamy and past betrayals as barriers (48, 49).

Within ongoing partnerships, relationship quality strongly influenced sexual wellbeing: affection and companionship often took priority when sexuality declined, providing support for life stressors (48, 53), whereas partners’ sexual problems, poor communication, conflicts, or stress negatively affected sexual expression (52, 53). Many women emphasised tolerance, compassion, and resilience in sustaining long-term bonds.

Relationships were also perceived as a coping strategy, with adaptations such as prioritising love, companionship, and commitment, helping sustain sensuality and intimacy in later life. Physical closeness (e.g., hugging) or adapted sexual behaviour, including less penetrative sex, and more masturbation, or oral sex, allowed intimacy to remain central, without sexual inactivity being problematic (48, 49, 53). Sexual activity was further described as reinforcing self-esteem, attractiveness, and belonging, highlighting its relational value (53).

### Theme 3: interventions and barriers to care

Varied strategies support sexual wellbeing, spanning medical, psychotherapeutic, and relational domains. Though trust in pharmaceutical solutions varied, they were reported as effective in addressing menopausal symptoms. Psychological and relational interventions were described as supporting communication, flexibility in sexual scripts, and reduce distress. However, stigma and limited services often constrained access, and women emphasised the need for integrated, age-sensitive approaches. These accounts underscore the importance of interventions addressing physical and relational aspects of later-life sexuality.

#### Self-esteem and body

Negative body image in later life could be eased by shifting focus from external pressure to appear “sexy” toward valuing health, capability, and self-expression. Many women fostered dignity and acceptance by emphasising what they could control, such as cleanliness, and functional wellbeing (54). Confidence grew through self-acceptance and less comparison with others. Interventions promoting self-esteem and sensuality, such as salsa classes, were effective (56). These inexpensive, accessible activities offered social connection, physical fitness, and safe spaces to embrace body movement and dress associated with pleasure, ultimately challenging age-related stereotypes and counteracting negative associations with ageing (56). Women could thus regain or sustain a sense of respectful, “age-appropriate” femininity.

#### Practical and physical barriers to accessing care

Women experienced multiple barriers to accessing appropriate care. Within families, health was rarely discussed, especially regarding intimate body parts considered “out of sight, out of mind” (50). Competing commitments made specialist appointments difficult, while stressful booking processes discouraged attendance, leading some to prefer alternatives such as consultant pharmacists. Screenings were often described as uncomfortable or invasive, with chronic illness, reduced mobility, and vaginal dryness compounding the pain. Distress was heightened when practitioners misinterpreted discomfort as failure to relax rather than recognising ageing or menopausal changes (50, 51). Sexual health services were perceived as youth-focused and embarrassing for older women to access (49, 55), reinforcing exclusion from care.

#### Systemic and relational barriers within healthcare

Once women accessed treatment, they faced systemic and relational barriers to appropriate care. Many felt judged for lifestyle choices (e.g., smoking) and described being deprioritised within the NHS (50, 55). Sexual problems were seen as taken less seriously for women, with partners offered medicalised solutions for erectile dysfunction while women rarely received specialist referrals; when they did, options such as Psychosexual and Relationship Therapy (PRT) were poorly explained and unsupported (48). This highlights perceptions of the NHS as ageist and disease-focused, with little attention to female sexuality beyond reproduction or STIs (51, 55). Despite positive outcomes when hormone therapy was provided (55), women viewed it as treatment for menopause rather than sexual dysfunction. A sense of invisibility grew after 50, as cervical screening declined and sexuality-related questions remained absent from NHS health checks, giving older women limited opportunities to discuss sexual problems. Women called for women-led, nurse-led, and age-specific clinics, alongside better staff training to normalise later-life sexuality and support diverse expressions of wellbeing (51, 55).

### Ageing women's sexuality: an integrated holistic experience

**Figure 1:**
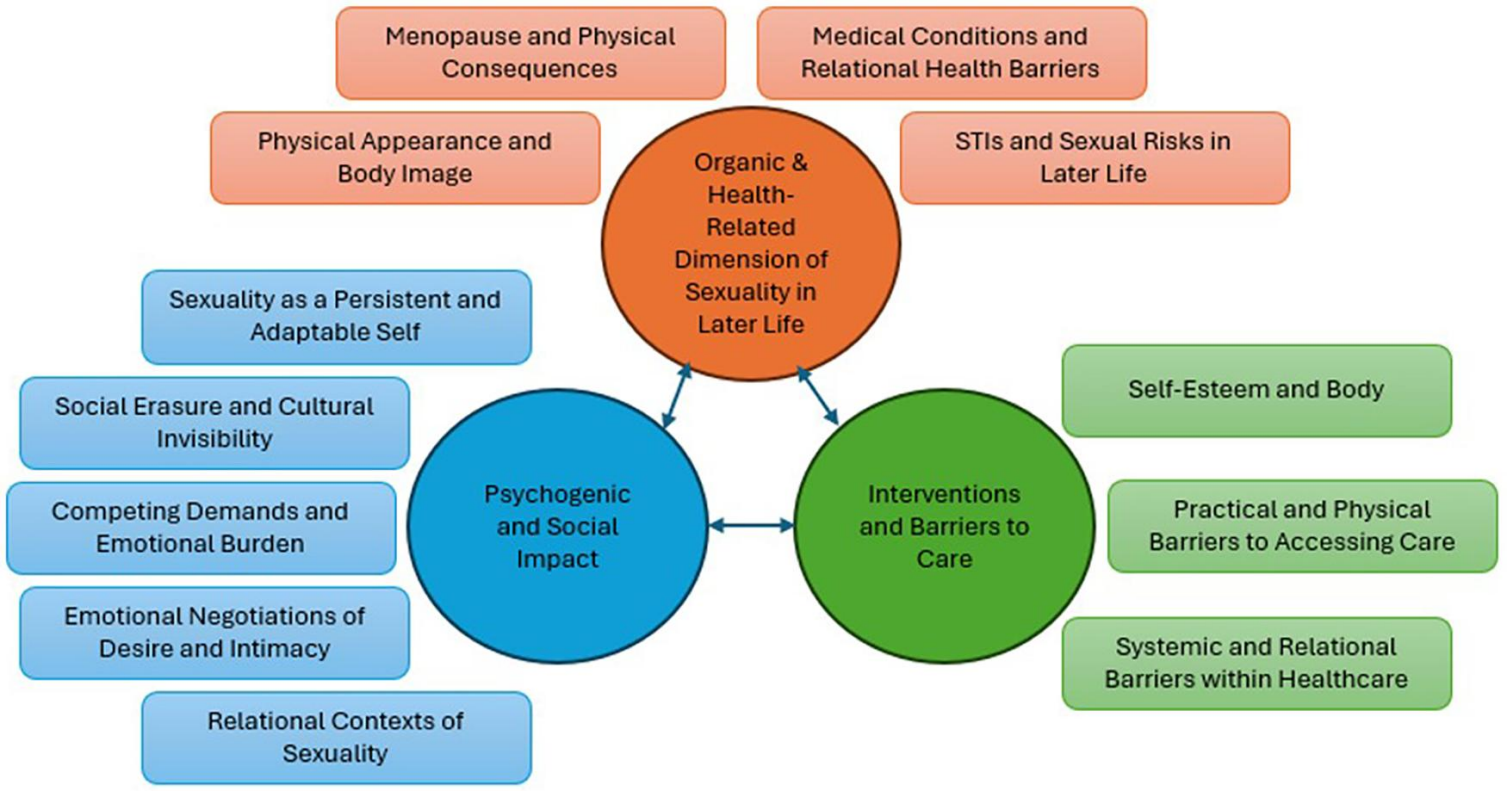
Interlinked themes in ageing women's sexuality.

Overall, the three themes cannot be viewed in isolation. The biological and health-related aspects are experienced alongside the psychological, social, and cultural influences, while interventions and barriers to care both shape and are shaped by these realities. These dimensions overlap and reinforce one another, together forming a holistic picture of how ageing women experience sexuality in later life (Figure 1).

Illustrative quotes for each subtheme in Table 1.

**Table 1:** Illustrative quotes for themes and subthemes.

Theme	Subtheme	Quotes
Organic & health-related dimensions of sexuality in later life	Menopause and physical consequences	“Since the menopause, an extremely important part of my life, intercourse, is ruined. This is because of vaginal dryness and spasm, reduction in physical desire (but not mental), and change and huge reduction in gaining orgasm and in intensity of orgasm” (55 y; inactive). (49) “I have always had a very active, healthy and very regular sex life until menopause, when my desire has plummeted, but not physical ability. GP says I have the need of HRT, and I am currently taking a herbal supplement (3 months) which does seem to be working and I do not believe it to be placebo.” (50–59y). (48) “Well, if people have got problems […] they think, oh it's all part and parcel…it's just my age.” (76y). (51) “Although we do not now have sexual relations or activity together, we do have lots of kisses, cuddles, laughter and discussions about all sorts of things, and I find this very necessary to our health and well-being.” (75–79 y). (52)
	Medical conditions and relational health barriers	“My husband has Parkinsons disease and TB of the vertebrae. At the age of 77 he also has dementia and is in hospital at this present time due to a fall” (73y; inactive). (49) “My husband is taking tablets which may or may not make him impotent. At 75 he thinks it's not necessary to discuss that with his GP [primary care physician], I disagree” (68y; inactive). (49) “Were just tired, I'd rather sit in front of the telly with a glass of wine and fall asleep in the armchair, that's us.” (53)
	Physical appearance and body image	“When you get older and everything starts flooding back into a very female-looking, soft body, I found that quite difficult. I used to keep quite muscular in a way, because I did a lot of gardening and keeping fit, and now I look down and I've got these rolls of fat and I think, ‘Oh, what is it, because I'm not fat, but what is it?’ You know? So, I find that quite difficult. I don't find the mirror quite as pleasing as I used to.” (54) “My concerns/worries have been to do with self-confidence—as I am getting older and fatter. My partner is wonderful and reassures me always that he sees me as he always did and loves me as I am, but I am a little more self-conscious. There is no reduction in his desire for me—it is only in my head. Weirdly, I am not affected by his changes in body shape and ageing. I love and want him just as much, so I can't reason out my own feelings. It has not had a negative impact on our sex life, it's the worried/concern questions that reflect my self esteem as I get older perhaps. (55–59 y)” (52)
	STIs and sexual risks in later life	“…the chances of getting pregnant are virtually nil aren't they? So from that point of view I look at it in a positive light.” (53)
Psychogenic and sociocultural impact	Sexuality as a persistent and adaptable self	“I have been on my own for 18 years, therefore without sex. I don't miss sex, I don't think about it, and I am quite content leading a single life” (51y; inactive). (49) My husband died [5–10] years ago, I am now with a new partner, although sex with my husband was fine. I found sex with my new partner much more uninhibited and more relaxed. (65–69y).” (52) “Sex has always been very important for me. I enjoyed masturbation but found it difficult to climax with a partner. With age the desire for sex has decreased but I would welcome any help. (As men can take Viagra.) (60–69y)” (48) “It's quite difficult. I want to wear a mask that shows what I'm really like inside. And then when people stand up for you on the tram and you think, ‘Well actually I'm probably more fit than you are,’ but because I've got a few wrinkles you know, that does it.” (69y) (54)
	Social erasure and cultural invisibility	“We have so many more freedoms than we did, and those freedoms, rather than us genuinely enjoying them, have been exploited to make us paranoid and miserable. It's become another thing to beat ourselves with as opposed to celebrate” (59y). (51) “when you're young you see people that are older, they're just old people, you don't think of them as having sexual feelings because they're not beautiful and attractive or whatever.” (67y). (54) “I like the fact that some companies are now using older models for clothes and so on. But those models are not typical in any way.” (79y). (54) “If you saw two old grey people snogging and fondling each other in the street, how would that go down? They'd think there was something wrong with us, wouldn't they?” (67y). (54)
	Competing demands and emotional burden	“It's our age group isn't it, people in their fifties…other people at work have the same issue, trying to care for parents when they're getting to an age where their capacity is diminishing and they're hitting their own health problems.” (53)
	Emotional negotiations of desire and intimacy	“Following breast cancer of [2–5] years ago, I have definitely found I have been less concerned about sexual relations. Sometimes I feel guilty as I know my husband would wish to have sexual relations more often. We do kiss and cuddle a lot and are very loving towards one another, which for me is usually satisfying.” (70–74y). (52) “I indulge in sexual activity to please my husband. I do not feel any sexual satisfaction from the activity. I do however enjoy the closeness and I am pleased that my husband enjoys the activity” (67 y; active). (49) “My husband has erection difficulties but is reluctant to try Viagra or similar, this has led to a lack of intimacy and some loss of respect.” (50–59y). (48)
	Relational contexts of sexuality	“I think sexual activities and sexual relationships become less important as you grow older. Love and friendship are far more important for wellbeing.” (60–64y). (52) “Oh yeah, we shagged this morning’ (laughs). I suppose yeah, absolutely, you feel good about yourself, don't you, you feel attractive, it's like a feeling of belonging to something special. Relationships are special.” (53)
Interventions and barriers to care	Self-esteem and body	“I just want my body to be functioning now. I don't want to be concerned with make-up and dressing in a young way. I want to look well-groomed, not fashionable for going out … I'm more concerned about being flexible. That's why I do yoga and I want to be fit. I'm not at all concerned about my type of swimming costume now but I would have been, you know, [whether it] looks good on me. Those things are not really important.” (77y). (54) “It's been really important, its changed how I buy clothes, its changed my shape you know, it's changed my fitness level, its, I've started meeting people again. It's an opportunity of learning like that, how to meet people and um what happens, and the rules and you know what touching means, what it means when people say certain things, how they look at you, it's been yeah it's been its all that …” (56)
	Practical and physical barriers to accessing care	“If you don't discuss sex as a, as a family between women, you may not discuss smears. So actually it becomes something that nobody really talks about … And then if nobody talks about it then nobody really sort of persuades you that it's a good idea.” (50) “Never mind getting the appointment, never mind actually on the bed and doing what you need to do … it is a barrier, the stress of having to check in … oh, I feel it drags me down … the whole procedure of “Reception.” (50) “Ladies of a certain age might think to themselves it was an abusive experience, that could be a reason why some women are reluctant to go these days … I was terrified.” (50) “I had a bad experience, just after I was fifty. I went through quite an early menopause, and then — do they call it vaginal atrophy? … I went for my smear test, the lady that did it wasn't very sympathetic and it was awful … she said it was my fault because I wasn't relaxed … I was very, very sore. I was very, very upset … I thought in five years … I'll have got over it, but when the five years came I just didn't go back.” (50)
	Systemic and relational barriers within healthcare	“I feel I'm judged … Am I doing this? Am I doing that? … Bloody hell, there's no hope for me really, is there?” (50) “NHS [National Health Service] seem reluctant to help with sexual problems in someone of our age. Penetrative sex is incredibly painful and I have been advised due to age.” (60–69y). (48) “Obviously, you go for your [cervical] smear test and all the rest of it, and then they don't…and even that, you come to an age and they say ‘right, you don't have them anymore’ and I don't think that's right.” (66y). (51)

## Discussion

This review synthesises UK qualitative evidence showing that later-life sexuality is best understood as a biopsychosocial phenomenon rather than a marker of biomedical decline. The findings highlight how embodied changes intersect with relational context and structural conditions (including service availability and age-related assumptions), shaping not only sexual activity but women's sense of identity and wellbeing. Interventions appeared most acceptable when they addressed these domains together, yet access remained uneven.

### Previous research

These findings align with earlier large-scale surveys showing continued, though less frequent, sexual activity in later life (57, 58). However, this review extends this evidence by centring women's lived experiences, which highlighted sexuality not simply as activity but as an enduring sense of self, relational connection, and vitality, even amid physical limitations. Whereas prior reviews criticised binary classifications of “active” vs. “inactive” (59), the women in this review demonstrated how sexuality evolved in diverse forms, including solo and non-partnered expressions, resisting reductive labels.

Stereotypes of older people as asexual, long recognised as barriers (60), were reconfirmed in here: women described how these assumptions discouraged disclosure of needs. In return, this reinforced healthcare providers’ reluctance to address sexual wellbeing (21).

Yet, the findings show that women also actively resisted these narratives by adapting sexual expression outside of traditional partnerships or through ongoing solo practices, aligning with prior research (61–63). This synthesis adds that these choices were often framed not only as practical adaptations but also as strategies of agency in the face of caregiving burdens and disproportionate gendered expectations. These pressures echoed Dennerstein's (64) “daily hassles” but were intensified in this generation by “double caring duties” and midlife economic insecurity (65).

Emotional negotiations of intimacy also reflected cultural scripts. Women frequently prioritise partners’ needs despite pain or lack of desire (66–68), shaped by coital norms in heterosexual relationships (69). The findings further suggest that these negotiations are ambivalent; valued as expressions of care but also sources of guilt, sadness, and frustration, illustrating the trade-offs that quantitative surveys often obscure.

The theme of subjective age further enriches this synthesis. Women's accounts of feeling younger than their chronological age resonated with concepts of an “ageless self” (70, 71). However, this review highlighted a persistent tension between this internal vitality and external pressures to “age successfully” by appearing youthful, reinforced by idealised media portrayals, thereby echoing previous research (72, 73).

Finally, while some scholars reframe ageing positively as rejecting the “male gaze”, defining femininity and sexuality beyond objectification (74, 75), or embracing “sexual wisdom”, where ageing fosters self-discovery, resilience, as a resource for resilience and intimacy (76), this review shows that many women themselves are already negotiating this shift. For some, later life created opportunities to be more disinhibited, focus on relational depth, or adapt practices to changing bodies, thereby challenging binaries of success vs. decline (72).

### Clinical implications

A clear application of these findings involves developing integrative therapeutic approaches looking at ageing women's sexuality holistically. Reflecting women's accounts of adapting to embodied and relational change, ageing brings inevitable changes to women's sexual lives, creating what Harris (77) describes in Acceptance and Commitment Therapy (ACT) as a “reality slap,” where expectations collide with embodied and relational realities.

The therapeutic task, therefore, lies in opening to this pain while reconnecting with values around intimacy, pleasure, and identity. In line with findings on internalised ageism and narrow sexual norms, for ageing women, this means fostering psychological flexibility by accepting unavoidable bodily and relational change and defusing from internalised ageist and sexual norms. This is further supported by cultivating present-moment, embodied awareness of pleasure and connection, alongside values clarification around intimacy to guide committed action that is value-consistent, rather than striving for unattainable ideals of youth or sexual “normality”.

This process can be strengthened through Solution-Focused Therapy, which supports small, concrete steps towards managing competing demands and cultivating hope for desired futures (78). Where women described relationship dynamics and gendered role pressures shaping sexual wellbeing, relationship therapy may be required to renegotiate dynamics, address disproportionate caring burdens, or explore unmet needs.

Beyond dialogue-based approaches, embodied and creative practices offer powerful avenues for reframing sexuality and sensuality. Building on accounts that confidence, self-image, and social belonging influence sexual wellbeing, movement-based group activities, such as salsa (56) aligning with a recent intervention involving burlesque workshops (79), provide safe, age-appropriate spaces where women can reconnect with their bodies, rediscover sensuality, and build confidence and social belonging. Taken together, such integrative interventions, linking ACT's psychological flexibility, solution-focused strategies, relational therapies, and embodied practices, can support women in embracing sexuality and intimacy as salient, evolving aspects of ageing.

Clinically, women's ambivalence toward medical treatments reflects a wider scepticism of pharmaceutical approaches to sexual dysfunction. Consistent with the synthesis highlighting uncertainty, mistrust, and uneven access to treatment, hormone therapies and oestrogen creams are effective for managing genitourinary syndrome of menopause (GSM) and dyspareunia (80–82), however, uptake remains limited due to mistrust and inconsistent provision. A key barrier is that providers often fail to initiate conversations about sexuality (21), reinforcing the invisibility of older women's sexual health. This can compound patient-reported barriers to disclosure and help-seeking. Addressing this requires systemic reform: first, sexual wellbeing should be incorporated into routine NHS Health Checks, which currently focus only on cardiovascular and metabolic risk, to normalise later-life sexuality and embed it within preventative care pathways. Second, services must be age-sensitive, women-led, and nurse-led, reducing stigma while promoting a holistic model of care that integrates medical, psychotherapeutic, and relational support beyond narrow disease-focused approaches. To achieve this, both specialist and primary care staff require further training and organisational support that not only improves knowledge but also tackles reluctance and stigma (e.g., discomfort, fear of offending, and assumptions of asexuality in older women), and provides practical tools, time, and referral pathways to initiate and respond to sexual wellbeing concerns.

### Strengths and limitations

This review's strengths lie in its focused scope and thematic clarity, drawing on peer-reviewed recent (2015 onwards) studies that capture evolving sociocultural attitudes through experiences. By adopting a narrative synthesis, the review was able to integrate organic, psychogenic, and treatment-related domains into a holistic account, holistically reflecting the WHO's definition of sexual health as encompassing pleasure, intimacy, and identity, not just reproduction (7).

Several limitations must be acknowledged. Sampling lacked diversity: most participants were white, heterosexual, cisgender, educated, and digitally literate (skewing towards “young old” women), with few women over 80 and little representation of disability, ethnic, religious, or sexual and gender minorities, restricting generalisability. Many studies focused on partnered, heterosexual, monogamous women, overlooking polyamorous relationships and underexploring the influence of relational status on health-related sexual wellbeing. Methodological tools such as the Female Sexual Function Index (FSFI) privileged intercourse-based measures, neglecting broader forms of intimacy (e.g., affection, companionship, non-penetrative practices) valued by participants, thereby narrowing definitions of sexual wellbeing.

### Future research

Future research must first expand to include more diverse populations and contexts, including transgender women, non-binary individuals, and those in polyamorous, casual, or solo relationships. Greater attention to intersectionality including ethnicity, disability, religion, socioeconomic status, and women aged 80+ would generate a more inclusive and representative evidence base, capturing the realities of ageing across varied social and cultural settings.

Building on this, there is a pressing need to design, test, and implement holistic integrative intervention frameworks that combine medical, psychotherapeutic, and relational approaches. Future studies should evaluate these models within real-world healthcare systems, addressing barriers such as stigma, staff training, and accessibility. More generally, testing the impact of embedding sexual wellbeing into policy, service design, and everyday consultations would ensure that research moves beyond identifying challenges to delivering practical, evidence-based solutions that genuinely improve later-life sexual health.

## Conclusion

This review demonstrates that ageing women's sexuality cannot be reduced to biological decline. It is sustained through a dynamic interplay of bodily change, psychological adaptation, and sociocultural negotiation. Women's accounts reveal sexuality as a source of identity, intimacy, and resilience, even as stigma, gendered expectations, and systemic neglect continue to constrain its expression. Despite limited and uneven access to holistic care, women showed creativity in adapting sexual practices and redefining pleasure beyond conventional norms. To support healthy ageing, sexual wellbeing must be recognised as integral to women's overall health, requiring inclusive, age-sensitive, and interdisciplinary approaches.
