# Supplementary material for: The qualitative experience of sexuality in ageing women: a narrative review
Source: Front Glob Womens Health. 2026 Mar 13;7:1793107. doi: 10.3389/fgwh.2026.1793107 (PMC13021586; doi:10.3389/fgwh.2026.1793107)
Supplement: Supplementary file 1 [file Table1.docx]

# Appendices

## Appendix 1: Database searches

### Appendix 2a: Search summary

| **Database** | **Platform** | **Results before duplicates removed** | **Results after duplicates removed** | **Date of search** |
| --- | --- | --- | --- | --- |
| MEDLINE® ALL | Ovid <1946 to August 08, 2025> | 89 | 87 | 11 Aug 2025 |
| APA PsycInfo® | ProQuest (1806 – current) | 181 | 168 | 11 Aug 2025 |
| Total number of results retrieved | | 270 |  |  |
| Number of duplicates removed | | 15 |  |  |
| Additional non-UK papers removed | | 70 |  |  |
| **Total number of results to screen** | |  | 185 |  |

### Appendix 2b: Search Strategy

| **Platform and database**: Ovid MEDLINE(R) ALL <1946 to August 08, 2025> | | |
| --- | --- | --- |
| 1 | *Women/ | 11701 |
| 2 | (female* or woman or women).ti,ab. | 2716198 |
| 3 | (Menopaus* or perimenopaus* or peri-menopaus* or postmenopaus* or post-menopaus* or climacterium or climacteric).ti,ab. | 116062 |
| 4 | *Menopause/ or *Perimenopause/ or *Postmenopause/ | 31132 |
| 5 | 1 or 2 or 3 or 4 | 2744906 |
| 6 | *Sexuality/ or exp *Sexual Behavior/ or *Sexual Health/ | 81022 |
| 7 | Sexuality.ti,ab. | 19520 |
| 8 | (sex* adj2 (activit* or expression or satisfaction or experience* or function or dysfunction or interest* or health or intercourse or desire)).ti,ab. | 86501 |
| 9 | 6 or 7 or 8 | 154170 |
| 10 | 5 and 9 | 62561 |
| 11 | (national health service* or nhs).ti,ab,in. | 318061 |
| 12 | (english not ((published or publication* or translat* or written or language* or speak* or literature or citation*) adj5 english)).ti,ab. | 148218 |
| 13 | (gb or "g.b." or britain* or (british* not "british columbia") or uk or "u.k." or united kingdom* or (england not "new england") or northern ireland* or northern irish* or scotland* or scottish* or ((wales or "south wales") not "new south wales") or welsh*).ab,in,jw,ti. | 2699459 |
| 14 | (bath or "bath's" or ((birmingham not alabama*) or ("birmingham's" not alabama*) or bradford or "bradford's" or brighton or "brighton's" or bristol or "bristol's" or carlisle* or "carlisle's" or (cambridge not (massachusetts* or boston* or harvard*)) or ("cambridge's" not (massachusetts* or boston* or harvard*)) or (canterbury not zealand*) or ("canterbury's" not zealand*) or chelmsford or "chelmsford's" or chester or "chester's" or chichester or "chichester's" or coventry or "coventry's" or derby or "derby's" or (durham not (carolina* or nc)) or ("durham's" not (carolina* or nc)) or ely or "ely's" or exeter or "exeter's" or gloucester or "gloucester's" or hereford or "hereford's" or hull or "hull's" or lancaster or "lancaster's" or leeds* or leicester or "leicester's" or (lincoln not nebraska*) or ("lincoln's" not nebraska*) or (liverpool not (new south wales* or nsw)) or ("liverpool's" not (new south wales* or nsw)) or ((london not (ontario* or ont or toronto*)) or ("london's" not (ontario* or ont or toronto*)) or manchester or "manchester's" or (newcastle not (new south wales* or nsw)) or ("newcastle's" not (new south wales* or nsw)) or norwich or "norwich's" or nottingham or "nottingham's" or oxford or "oxford's" or peterborough or "peterborough's" or plymouth or "plymouth's" or portsmouth or "portsmouth's" or preston or "preston's" or ripon or "ripon's" or salford or "salford's" or salisbury or "salisbury's" or sheffield or "sheffield's" or southampton or "southampton's" or st albans or stoke or "stoke's" or sunderland or "sunderland's" or truro or "truro's" or wakefield or "wakefield's" or wells or westminster or "westminster's" or winchester or "winchester's" or wolverhampton or "wolverhampton's" or (worcester not (massachusetts* or boston* or harvard*)) or ("worcester's" not (massachusetts* or boston* or harvard*)) or (york not ("new york*" or ny or ontario* or ont or toronto*)) or ("york's" not ("new york*" or ny or ontario* or ont or toronto*))))).ti,ab,in. | 1971351 |
| 15 | (bangor or "bangor's" or cardiff or "cardiff's" or newport or "newport's" or st asaph or "st asaph's" or st davids or swansea or "swansea's").ti,ab,in. | 80286 |
| 16 | (aberdeen or "aberdeen's" or dundee or "dundee's" or edinburgh or "edinburgh's" or glasgow or "glasgow's" or inverness or (perth not australia*) or ("perth's" not australia*) or stirling or "stirling's").ti,ab,in. | 289829 |
| 17 | (armagh or "armagh's" or belfast or "belfast's" or lisburn or "lisburn's" or londonderry or "londonderry's" or derry or "derry's" or newry or "newry's").ti,ab,in. | 39072 |
| 18 | 11 or 12 or 13 or 14 or 15 or 16 or 17 | 3330373 |
| 19 | (exp africa/ or exp americas/ or exp antarctic regions/ or exp arctic regions/ or exp asia/ or exp oceania/) not (exp great britain/ or europe/) | 3604346 |
| 20 | 18 not 19 | 3110131 |
| 21 | 10 and 20 | 6255 |
| 22 | "Document Analysis"/ or focus groups/ or interviews as topic/ or narration/ or qualitative research/ | 194044 |
| 23 | ((depth or face or group or guided or indepth or informal or semistructured or structured or unstructured) adj4 (discussion or discussions or interview or interviewed or interviews or questionnaire or questionnaires)).ti,ab,kf,kw. | 259656 |
| 24 | (ethnographic or ethnography or (field adj1 work) or fieldwork or (focus adj1 (group or groups)) or (groups adj2 interviewed) or (key adj1 (informant or informants)) or (qualitative adj2 (research or studies or studies or synthesis))).ti,ab,kf,kw. | 159596 |
| 25 | 22 or 23 or 24 | 435347 |
| 26 | 21 and 25 | 593 |
| 27 | limit 26 to (english language and yr="2015 -Current" and ("middle age (45 to 64 years)" or "middle aged (45 plus years)" or "all aged (65 and over)" or "aged (80 and over)")) | 89 |
| **Platform and database**: Ovid APA PsycInfo <1806 August 2025 Week 1> | | |
| 1 | *Human Females/ | 60470 |
| 2 | (female* or woman or women).ti,ab. | 690355 |
| 3 | (Menopaus* or perimenopaus* or peri-menopaus* or postmenopaus* or post-menopaus* or climacterium or climacteric).ti,ab. | 7729 |
| 4 | *Menopause/ | 3772 |
| 5 | 1 or 2 or 3 or 4 | 696800 |
| 6 | *Sexuality/ or *Psychosexual Behavior/ | 37981 |
| 7 | Sexuality.ti,ab. | 32598 |
| 8 | (sex* adj2 (activit* or expression or satisfaction or experience* or function or dysfunction or interest* or health or intercourse or desire)).ti,ab. | 50284 |
| 9 | 6 or 7 or 8 | 88711 |
| 10 | 5 and 9 | 37432 |
| 11 | (national health service* or nhs).ti,ab,in. | 32456 |
| 12 | (english not ((published or publication* or translat* or written or language* or speak* or literature or citation*) adj5 english)).ti,ab. | 105824 |
| 13 | (gb or "g.b." or britain* or (british* not "british columbia") or uk or "u.k." or united kingdom* or (england not "new england") or northern ireland* or northern irish* or scotland* or scottish* or ((wales or "south wales") not "new south wales") or welsh*).ab,in,jx,ti. | 567193 |
| 14 | (bath or "bath's" or ((birmingham not alabama*) or ("birmingham's" not alabama*) or bradford or "bradford's" or brighton or "brighton's" or bristol or "bristol's" or carlisle* or "carlisle's" or (cambridge not (massachusetts* or boston* or harvard*)) or ("cambridge's" not (massachusetts* or boston* or harvard*)) or (canterbury not zealand*) or ("canterbury's" not zealand*) or chelmsford or "chelmsford's" or chester or "chester's" or chichester or "chichester's" or coventry or "coventry's" or derby or "derby's" or (durham not (carolina* or nc)) or ("durham's" not (carolina* or nc)) or ely or "ely's" or exeter or "exeter's" or gloucester or "gloucester's" or hereford or "hereford's" or hull or "hull's" or lancaster or "lancaster's" or leeds* or leicester or "leicester's" or (lincoln not nebraska*) or ("lincoln's" not nebraska*) or (liverpool not (new south wales* or nsw)) or ("liverpool's" not (new south wales* or nsw)) or ((london not (ontario* or ont or toronto*)) or ("london's" not (ontario* or ont or toronto*)) or manchester or "manchester's" or (newcastle not (new south wales* or nsw)) or ("newcastle's" not (new south wales* or nsw)) or norwich or "norwich's" or nottingham or "nottingham's" or oxford or "oxford's" or peterborough or "peterborough's" or plymouth or "plymouth's" or portsmouth or "portsmouth's" or preston or "preston's" or ripon or "ripon's" or salford or "salford's" or salisbury or "salisbury's" or sheffield or "sheffield's" or southampton or "southampton's" or st albans or stoke or "stoke's" or sunderland or "sunderland's" or truro or "truro's" or wakefield or "wakefield's" or wells or westminster or "westminster's" or winchester or "winchester's" or wolverhampton or "wolverhampton's" or (worcester not (massachusetts* or boston* or harvard*)) or ("worcester's" not (massachusetts* or boston* or harvard*)) or (york not ("new york*" or ny or ontario* or ont or toronto*)) or ("york's" not ("new york*" or ny or ontario* or ont or toronto*))))).ti,ab,in. | 443432 |
| 15 | (bangor or "bangor's" or cardiff or "cardiff's" or newport or "newport's" or st asaph or "st asaph's" or st davids or swansea or "swansea's").ti,ab,in. | 23259 |
| 16 | (aberdeen or "aberdeen's" or dundee or "dundee's" or edinburgh or "edinburgh's" or glasgow or "glasgow's" or inverness or (perth not australia*) or ("perth's" not australia*) or stirling or "stirling's").ti,ab,in. | 55018 |
| 17 | (armagh or "armagh's" or belfast or "belfast's" or lisburn or "lisburn's" or londonderry or "londonderry's" or derry or "derry's" or newry or "newry's").ti,ab,in. | 7612 |
| 18 | 11 or 12 or 13 or 14 or 15 or 16 or 17 | 733467 |
| 19 | (africa or america or antarctic or arctic or asia or oceania) not ("great Britain" or europe).af. | 65342 |
| 20 | 18 not 19 | 724776 |
| 21 | 10 and 20 | 3947 |
| 22 | Focus Group/ or Focus Group Interview/ or Grounded Theory/ or Interpretative Phenomenological Analysis/ or Narrative Analysis/ or Qualitative Measures/ or Qualitative Methods/ or Semi-Structured Interview/ or Thematic Analysis/ | 23533 |
| 23 | (focus group or interview or qualitative study).md. | 559497 |
| 24 | ("Document Analysis" or focus groups or "interviews as topic" or narration or "qualitative research").mh. | 48790 |
| 25 | ((depth or face or group or guided or indepth or informal or semistructured or structured or unstructured) adj4 (discussion or discussions or interview or interviewed or interviews or questionnaire or questionnaires)).ti,ab,id. | 186471 |
| 26 | (ethnographic or ethnography or (field adj1 work) or fieldwork or (focus adj1 (group or groups)) or (groups adj2 interviewed) or (key adj1 (informant or informants)) or (qualitative adj2 (research or studies or studies or synthesis))).ti,ab,id. | 136654 |
| 27 | 22 or 23 or 24 or 25 or 26 | 679136 |
| 28 | 21 and 27 | 896 |
| 29 | limit 28 to (english language and ("360 middle age (age 40 to 64 yrs)" or "380 aged (age 65 yrs and older)" or "390 very old (age 85 yrs and older)") and yr="2015 -Current") | 181 |

## Appendix 2: Table of Study Characteristics for the nine selected studies

| Authors | Title | Year | Focus | Demographics | Method |
| --- | --- | --- | --- | --- | --- |
| Sarah Milton | Becoming more of myself': Safe sensuality, salsa and ageing | 2017 | This study explores how salsa dancing fosters safe expressions of sensuality and supports identity development in ageing women. | 20 women in their 50s; all heterosexual; 19 single/dating/new relationships vs 1 married; all White British. | Semi-structured interviews |
| Alison Bravington, Hong Chen, Judith Dyson, Lesley Jones, Christopher Dalgliesh, Amee Bryan, Julietta Patnick, Una Macleod | Challenges and opportunities for cervical screening in women over the age of 50 years: a qualitative study | 2022 | The study explores barriers and opportunities for improving cervical cancer screening among women aged 50–64, a group facing rising incidence but lower participation, by examining experiences of both practitioners and patients. | 24 women aged between 53 and 64; 6 single, 17 with male partner, 1 with female partner; 13 working, 14 retired or not working due to illness. | Semi-structured interviews |
| Hayley James, James Nazroo, Georgia Chatzi, Paul Simpson | How Do Women and Men Negotiate Sex in Later Life Relationships? A Qualitative Analysis of Data from the English Longitudinal Study of Ageing | 2023 | The study explores how older adults negotiate sexual activity and intimacy in later-life relationships. | 1,986 respondents, mostly aged between 60 and 69; mostly female; 88.6% heterosexual; majority partnered/cohabiting; largely White. | Qualitative data from survey (ELSA Wave 6) |
| Josie Tetley, David M. Lee, James Nazroo, Sharron Hinchliff | Let's talk about sex-what do older men and women say about their sexual relations and sexual activities? A qualitative analysis of ELSA Wave 6 data | 2018 | The study examines how older adults experience and negotiate sexual activity and intimacy in later life. | 668 women; 18 <50 years, 62 >80 years, 591 between 50 and 79; 410 married/co-habiting, 148 widowed, 112 separated/never married; self-rated health: 286 excellent/very good, 199 good, 186 fair/poor; 634 reported sexual experience (mostly opposite-sex). | Qualitative data from survey (ELSA Wave 6) |
| Sophie Patterson, kate Jehan | Love (and) ageing well: A qualitative study of sexual health in the context of ageing well among women aged 50 and over | 2024 | The study explores how women aged 50+ interpret and experience sexual health in the context of ageing well. | 21 women aged 50–79; 20 White British, 1 Asian British; all heterosexual; 2 perimenopausal, 19 postmenopausal; 10 retired, 6 in paid employment, 5 not working due to ill-health; 16 with children; relationship status: 9 in partnership, 9 with previous marriage, 3 never married/single. | Semi-structured interviews |
| Ruth Lewis, Kirstin R. Mitchell, Catherine H. Mercer, Jessica Datta, Kyle G. Jones, Kaye Wellings | Navigating new sexual partnerships in midlife: a socioecological perspective on factors shaping STI risk perceptions and practices | 2020 | The study explores how midlife adults navigate new sexual partnerships and perceive STI risk, using a socioecological framework to understand individual, relational, and societal influences. | 10 women; 8 aged 40–49 and 2 aged 50–59; 9 divorced/separated, 1 never married; 4 did not have a new sexual partner since last relationship; 5 in relationship/dating someone; 5 not interested in relationships; 8 had children living with participant; 1 has a child who does not live with participant; 1 never had children. | Semi-structured interviews |
| Lauren B. Towler, Cynthia A. Graham, F.L. Bishop, Sharron Hinchliff | Older adults' embodied experiences of aging and their perceptions of societal stigmas toward sexuality in later life | 2021 | This qualitative study explores how older adults experience their aging bodies and perceive societal stigmas surrounding sexuality in later life, using reflexive thematic analysis of in-depth interviews. | 16 women; no clear demographic distinction between male and female participants; overall 55% of participants were married, rest single/widowed; 27% still in work at time of interview. | Semi-structured interviews |
| Helena Harder, Rachel M. Starkings, Lesley J. Fallowfield, Usha Menon, Ian J. Jacobs, Valerie A. Jenkins | Sexual functioning in 4,418 postmenopausal women participating in UKCTOCS: a qualitative free-text analysis | 2019 | This study explored sexual functioning and experiences in postmenopausal women using qualitative analysis of free-text responses from 4,418 participants in the UKCTOCS trial. | 4,418 women aged between 50 and 74; 2,883 had an intimate partner; 995 reported being sexually active. | Qualitative free-text analysis from FSAQ questionnaire from UKCTOS |
| Kaye Wellings, Lorna Gibson, Ruth Lewis, Jessica Datta, Wendy Macdowall, Kirstin Mitchell | “We're Just Tired”: Influences on Sexual Activity Among Male-Partnered Women in Midlife; A Mixed Method Study | 2023 | The study examines factors affecting sexual activity among women aged 45–59 in the UK, highlighting the importance of lifestyle and relationship quality over menopausal status. | 23 women aged 45–59; partnered with men; menopausal status varied; some peri-menopausal, others post-menopausal; analysis from Natsal-3 survey + semi-structured interviews. | Semi-structured interviews of women who took part in the (Natsal-3, reportedly unsatisfied with their current sex life |

*Note*: ELSA = English Longitudinal Study of Ageing; UKCTOS = UK Collaborative Trial of Ovarian Cancer Screening; FSAQ = Fallowfield's Sexual Activity Questionnaire; Natsal-3 = National Study of Sexual Attitudes and Lifestyles
